# Vanadium oxide nanorods as an electrode material for solid state supercapacitor

**DOI:** 10.1038/s41598-022-25707-z

**Published:** 2022-12-05

**Authors:** Amrita Jain, Sai Rashmi Manippady, Rui Tang, Hirotomo Nishihara, Kamil Sobczak, Vlastimil Matejka, Monika Michalska

**Affiliations:** 1 Institute of Fundamental Technological Research, Polish Academy of Sciences, Pawińskiego 5B, 02-106 Warsaw, Poland; 2 Advanced Institute for Materials Research (AIMR-WPI), Tohoku University, 2-1-1 Katahira, Aoba-ku, Sendai, 980-8577 Japan; 3 Institute of Multidisciplinary Research for Advanced Materials, Tohoku University, 2-1-1 Katahira, Aoba-ku, Sendai, 980-8577 Japan; 4 Faculty of Chemistry, Biological and Chemical Research Centre, University of Warsaw, Zwirki i Wigury 101, 02-089 Warsaw, Poland; 5 Department of Chemistry and Physico-Chemical Processes, Faculty of Materials Science and Technology, VŠB-Technical University of Ostrava, 17 Listopadu 2172/15, 708 00 Ostrava-Poruba, Czech Republic

**Keywords:** Energy science and technology, Materials science, Nanoscience and technology

## Abstract

The electrochemical properties of metal oxides are very attractive and fascinating in general, making them a potential candidate for supercapacitor application. Vanadium oxide is of particular interest because it possesses a variety of valence states and is also cost effective with low toxicity and a wide voltage window. In the present study, vanadium oxide nanorods were synthesized using a modified sol–gel technique at low temperature. Surface morphology and crystallinity studies were carried out by using scanning electron microscopy, transmission electron microscopy, X-ray diffraction and X-ray photoelectron spectroscopy analysis. To the best of our knowledge, the as-prepared nanorods were tested with magnesium ion based polymer gel electrolyte for the first time. The prepared supercapacitor cell exhibits high capacitance values of the order of ~ 141.8 F g^−1^ with power density of ~ 2.3 kW kg^−1^ and energy density of ~ 19.1 Wh kg^−1^. The cells show excellent rate capability and good cycling stability.

## Introduction

As the population is growing, the energy demands are raising and it is difficult to fulfill/meet the requirement with the conventional energy sources such as fossil fuels, coal, etc., and hence the search for alternative new and environmentally benign energy storage systems is attracting the research community. In recent years, significant developments have been made in the field of lithium-ion batteries (LIBs), and it is one of the most widely used energy storage systems due to its advantages such as high energy density, long service life, low cost, and almost no memory effect^1,2^. However, along with these advantages, there is also a fact that lithium resources are limited in nature, and hence it gives a strong motivation to explore innovative materials with almost comparable results.

Supercapacitors, also called ultracapacitors, have the potential to become efficient energy storage devices with properties comparable to those of existing devices. Supercapacitors due to their fast charging and power delivery can complement or even surpass batteries in electrochemical energy storage and harvesting applications, where high power output is required^3–8^.

Recently, transition metal oxides such as Co_3_O_4_^9,10^, MnO_2_^11–13^, TiO_2_^14–16^, Fe_3_O_4_^17,18^, WO_3_^19,20^, NiO^21,22^, V_2_O_5_^23,24^ etc. have gained attention towards electrode materials in supercapacitors due to their high specific capacitance and energy density. Specially due to the high abundance, variable oxidation states and cost effectiveness, V_2_O_5_ is prone to be a promising electroactive redox material. Thanks to the high theoretical capacity (440 mAh g^−1^) arising from multi-valence properties of V_2_O_5_ to form not only a multi-step faradaic process, but also different morphologies and crystal structures^25,26^. Although researchers have focused on developing pseudocapacitive materials from their high specific capacitance obtained by faradaic reactions, their low electrical conductivity and cyclic stability are still the main challenges facing them.

Liquid electrolytes have played a very important and crucial role in energy storage devices rather than solid electrolytes, particularly supercapacitors because of their high ionic conductivity (10^–3^ to 10^–2^ S cm^−1^) and superior wettability of the electrolyte on an electrode, resulting in a fast charge–discharge rate and low interfacial resistance^27–31^. However, in addition to these benefits, electrochemical devices based on liquid electrolytes may pose major risks such as electrolyte leakage, toxicity, and fire explosion^32^. Additionally, because of its liquid nature, it is always a disadvantage that it is not possible to fabricate flexible devices. These challenges led the research community to develop gel polymer electrolytes (GPEs) as a substitute to a liquid electrolyte for supercapacitor application. The GPEs are typically prepared by entrapping a liquid electrolyte into a polymer matrix (e.g. polyvinyl alcohol (PVA), polyvinylidenefluoride-*co*-hexafluoropropylene (PVdF-HFP), polyethylene glycol (PEG) etc.)^33–35^. GPEs are attractive for energy storage applications due to their high ionic conductivity and electrochemical stability which are comparable to liquid electrolytes, in addition to the fact that GPE also offers dual functions, it can act as an electrolyte and also as a separator^36^.

In this work, we mainly focus on the chemical sol–gel synthesis of the V_2_O_5_ material to which the different compositions of complexing agent were selected and its application in a supercapacitor using gel polymer electrolyte using magnesium salt. Magnesium was chosen as a salt because it is divalent in nature, and because of this, a high specific capacity and energy density are possible. In conjunction with magnesium, PVdF-HFP is used as a polymer and propylene carbonate as the plasticizer. The so-prepared vanadium oxide materials were used as electrode materials. The materials were used to fabricate supercapacitor cells, and the overall performance of the cells was characterized by using impedance spectroscopy, cyclic voltammetry, galvanostatic charge–discharge technique and cyclic efficiency.

## Methods

### Preparation of V_2_O_5_ nanorods

**Figure 1:**
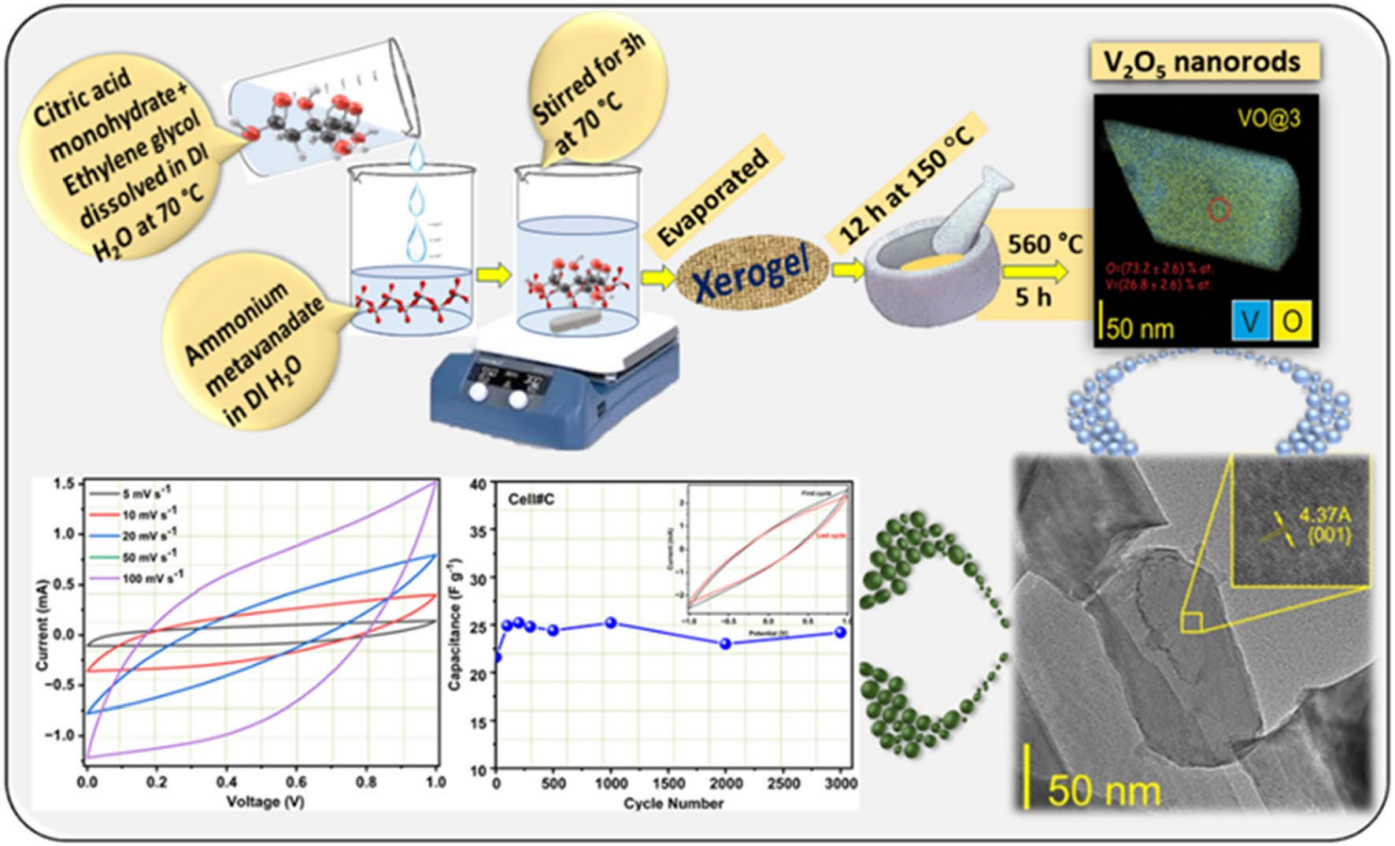
Schematic representation of the V_2_O_5_ nanorods preparation using different chelating agent.

All the chemicals were purchased from Merck and used without further purification. The vanadium oxide powders (V_2_O_5_) were synthesized using a modified sol–gel technique. Ammonium metavanadate (NH_4_VO_3_, 99.0%, Sigma-Aldrich) was first dissolved in deionized water for 2 h at 70 °C. Then, the solution of citric acid monohydrate C_6_H_8_O_8_ H_2_O (99.5%, Sigma-Aldrich) was added as the main chelating agent. Acetic acid (99.5%, Sigma-Aldrich) or ethylene glycol (99%, Sigma-Aldrich) was added as the second chelating agent. The solutions were then mixed and stirred for 3 h at 70 °C. To remove the residual water from the solutions and achieve a transparent gel, the evaporation process was applied. The xerogels (pre-heated powder) were dried in air for 12 h at 150 °C and ground in agate mortar. In the last step, the powders were heated in air from room temperature to 560 °C for 12 h and kept at 560 °C for 5 h. The sample obtained only using citric acid as the main chelating agent was labelled 'VO@1'. The powder synthesized with two chelating agents: citric with acetic acids and citric acid with ethylene glycol was labelled 'VO@2' and 'VO@3', respectively. Figure 1, shows the schematic representation of the synthesis process used in the present case.

### Instrumental details

Morphological studies of VO@1, VO@2 and VO@3 were conducted on a FEI Talos F200X transmission microscope at 200 kV. The measurements were performed in the TEM and STEM modes using high-angle annular dark-field imaging. Energy-dispersive X-ray spectroscopy (EDX) on a Brucker BD4 instrument was used for mapping the distribution of elements. The samples for the TEM observations were prepared by dropping the colloid particle dispersion on a carbon film supported on a 300-mesh copper grid. Scanning electron microscopy using the instrument SEM/FIB-Zeiss Crossbeam 350, Germany, and EDX were carried out using Ametek EDAX, Octane Elite. The accelerating voltage used for EDX elemental analysis was reduced to 7 kV minimizing the penetration depth. X-ray powder diffraction analysis was investigated on the X-ray diffractometer MiniFlex 600 (Rigaku, Japan) equipped with a Co tube operated at 40 kV and 15 mA, DteX Ultra 250 was used as a detector. The XRD patterns were collected in 10–60° 2θ range with a step of 0.01° and a scanning rate of 10°min^−1^. The recorded patterns were evaluated using the PDXL software (Rigaku, Japan) and the PDF 2 database, released in 2019. The specific surface areas of the samples (VO@1, VO@2, VO@3) were measured using AutoSorb IQ, Quantachrome, USA under nitrogen gas flow and the pore size distribution curves were obtained by using the DFT model. The surface chemical structure was analyzed by XPS (JEOL JPS-9200, operating at a pressure of 10^−7^ Pa with an Al Kα X-ray source generated at 10 kV and 10 A). The XPS spectra obtained were calibrated by the C 1 s peak position (284.5 eV).

### Fabrication and characterizations of supercapacitor cells

VO@1, VO@2 and VO@3 were used as an electrode materials for the fabrication of supercapacitor cells and GPE was used as an electrolyte material. In the present study, GPE was prepared by using the solution cast technique. The final optimized composition of the GPE used is PVdF-HFP-PC-Mg(ClO_4_)_2_. The preparation, characterization, and optimization of the films have been described elsewhere^37^. Electrodes were prepared in the form of planar sheets by using carbon cloth as the substrate material. Working electrodes were VO@1, VO@2 and VO@3 which were mixed with acetylene black as a conducting reagent and the polymer PVdF was used as a binder in the 80:10:10 (w/w) ratio and acetone was used as a solvent to form a slurry. The prepared slurry was coated on flexible carbon cloth (AvCarb, USA). The films were dried overnight at ~ 80 °C before being used as an electrode material. The mass of the active electrode material was between 0.7–0.9 mg and area of the electrode materials was 1.0 cm^2^. Three different supercapacitor cells (Cell#A–#C) were prepared by using VO@1, VO@2 and VO@3 as an electrode materials and gel polymer electrolyte PVDF-HFP-PC-Mg(ClO_4_)_2_ as an electrolyte material in a two-electrode configuration using Swagelok system which means gel polymer electrolytes were sandwiched in between the symmetrical films of VO@1, VO@2 and VO@3, respectively as a solid state device. The electrochemical cells were characterized by using various electrochemical techniques, such as electrochemical impedance spectroscopy (EIS), cyclic voltammetry (CV), and galvanostatic charge–discharge (GCD). All electrochemical measurements were performed using the Biologic VMP3 workstation.

## Results and discussion

### Structural and morphological properties

**Figure 2:**
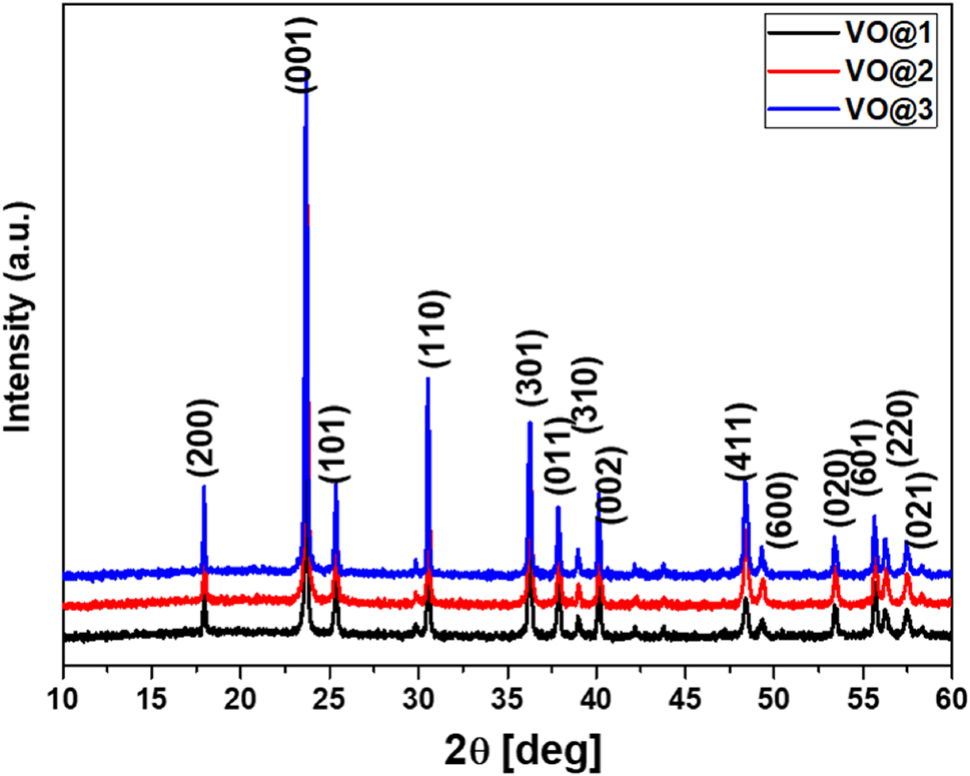
The XRD pattern of V_2_O_5_ powders obtained via modified sol–gel method using citric acid as the main chelating agent (**VO@1**) with: acetic acid (**VO@2**), ethylene glycol (**VO@3**) as the second chelating agent.

The XRD patterns of all vanadium oxide powders prepared from different precursors are shown in Fig. 2. All diffraction lines are indexed to the orthorhombic crystal structure (Shcherbinaite, syn, (space group: Pmmn)) of vanadium oxide and perfectly matched to the V_2_O_5_ diffraction lines from the PDF database (entry # 041-1426). Furthermore, the lattice refinement indicated that there were no significant differences in the crystal lattice parameters of the V_2_O_5_ phase. The unit cell parameters calculated in the PDXL software (Rigaku, Japan), according to the XRD data shown in Fig. 1 are listed in Table 1, and are consistent with the standard values for vanadium oxide (V_2_O_5_): a_0_ = 11.516 Å, b_0_ = 3.5656 Å, c_0_ = 4.3727 Å and V_0_ = 179.55 Å^3^. The average crystallite size of these V_2_O_5_ powders estimated by the Halder-Wagner method implemented in the PDXL II software (Rigaku, Japan), were between 57 and 77 nm.

**Table 1:** Crystallite sizes, lattice parameters of V_2_O_5_ powders.

Sample	d (nm)	a (Å)	b (Å)	c (Å)	V (Å^3^)
VO@1	66	11.515	3.5650	4.3746	179.58
VO@2	57	11.516	3.5653	4.3742	179.60
VO@3	77	11.515	3.5649	4.3734	179.52

**Figure 3:**
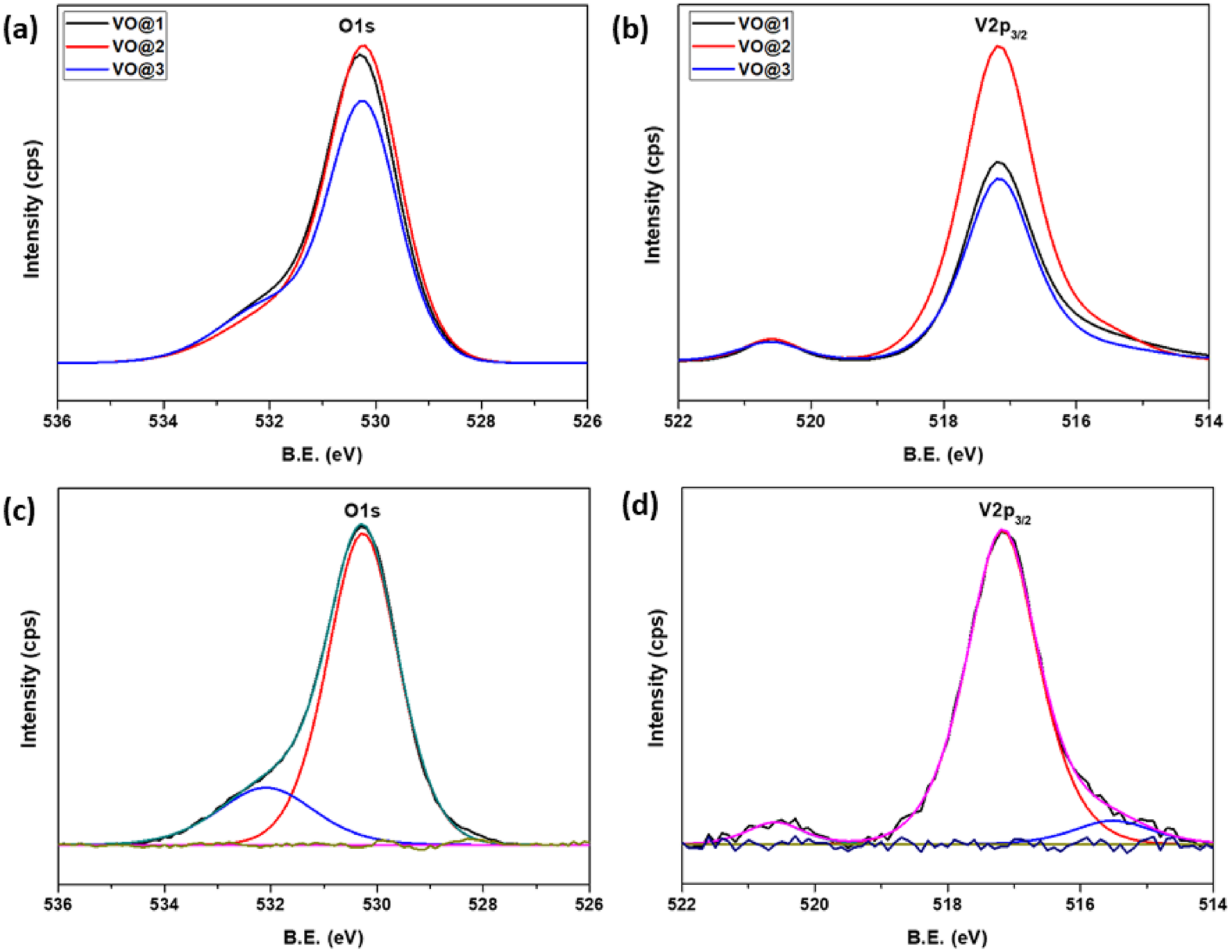
The normalized XPS spectra (**a**) O1s, (**b**) V2p_3/2_ of all V_2_O_5_ powders and (**c**) high-resolution spectra O1s, (**d**) V2p_3/2_ of VO@2 sample.

The normalized X-ray photoelectron spectroscopy spectrum presented in Fig. 3a,b revealed the presence of V and O elements of all analysed vanadium oxide nanocrystalline powders. The chemical states of the V_2_O_5_ elements (for a selected VO@2 sample) were evaluated by their high-resolution XPS spectra, as shown in Fig. 3c,d. Two characteristic lines, located at 530.2 eV and 517.2 eV observed for all samples correspond to O1s and V2p_3/2_ in vanadium pentoxide (V_2_O_5_)^38–41^.

**Figure 4:**
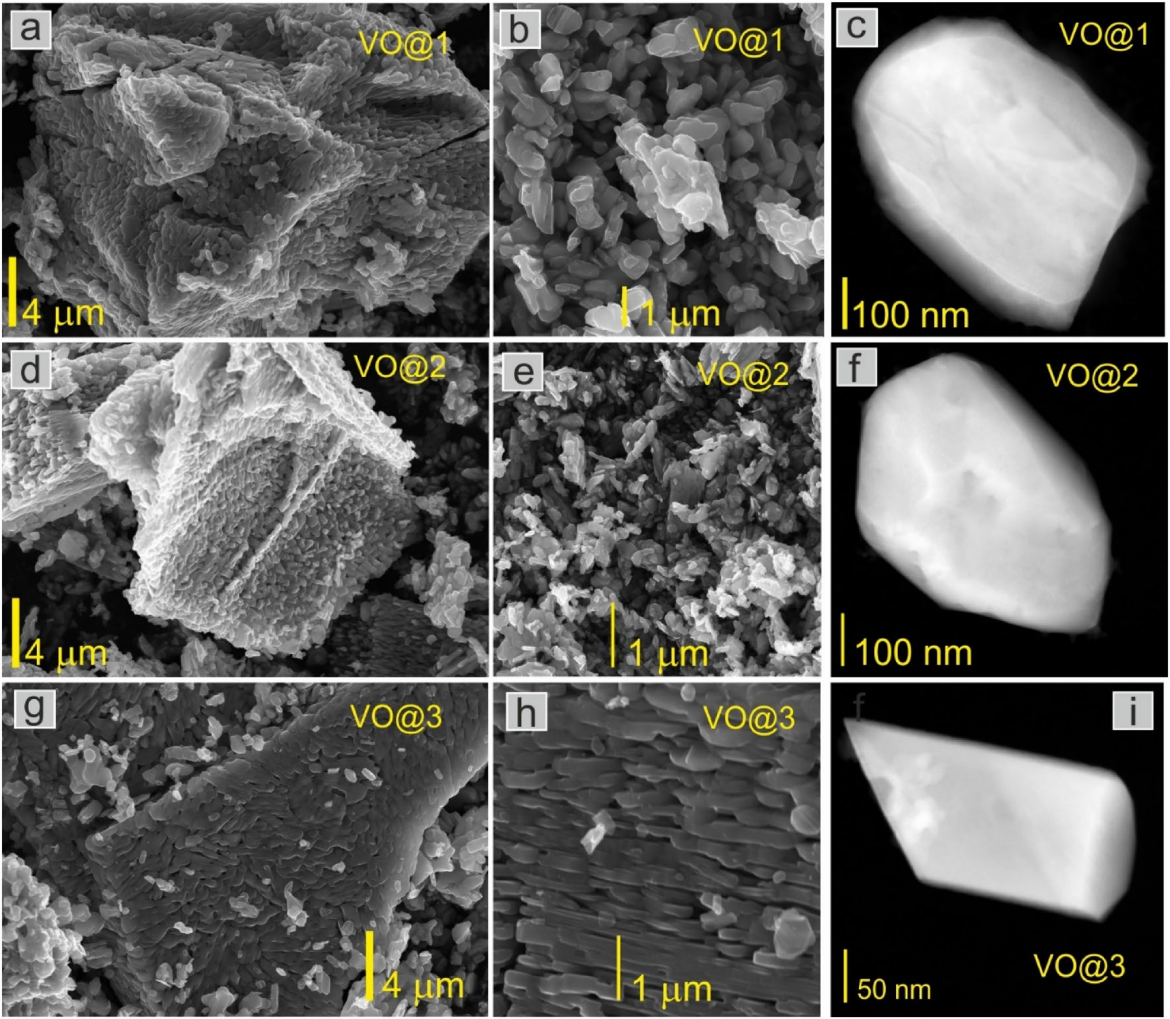
SEM and STEM images of V_2_O_5_ powders obtained using a modified sol–gel method using citric acid as the main chelating agent (**a**–**c**) with acetic acid (**d**–**f**), ethylene glycol (**g**–**i**) as the second chelating agent (figure **c**, **f** and **i** are STEM images).

The morphology of the investigated samples is presented in Fig. 4a,b,d,e,g,h where it can be seen that all powders possessed a nanorod shape with an average diameter of 500 nm to 1 μm, also in Fig. 4c,f,i the single nanograin for each sample was shown in the TEM image.

**Figure 5:**
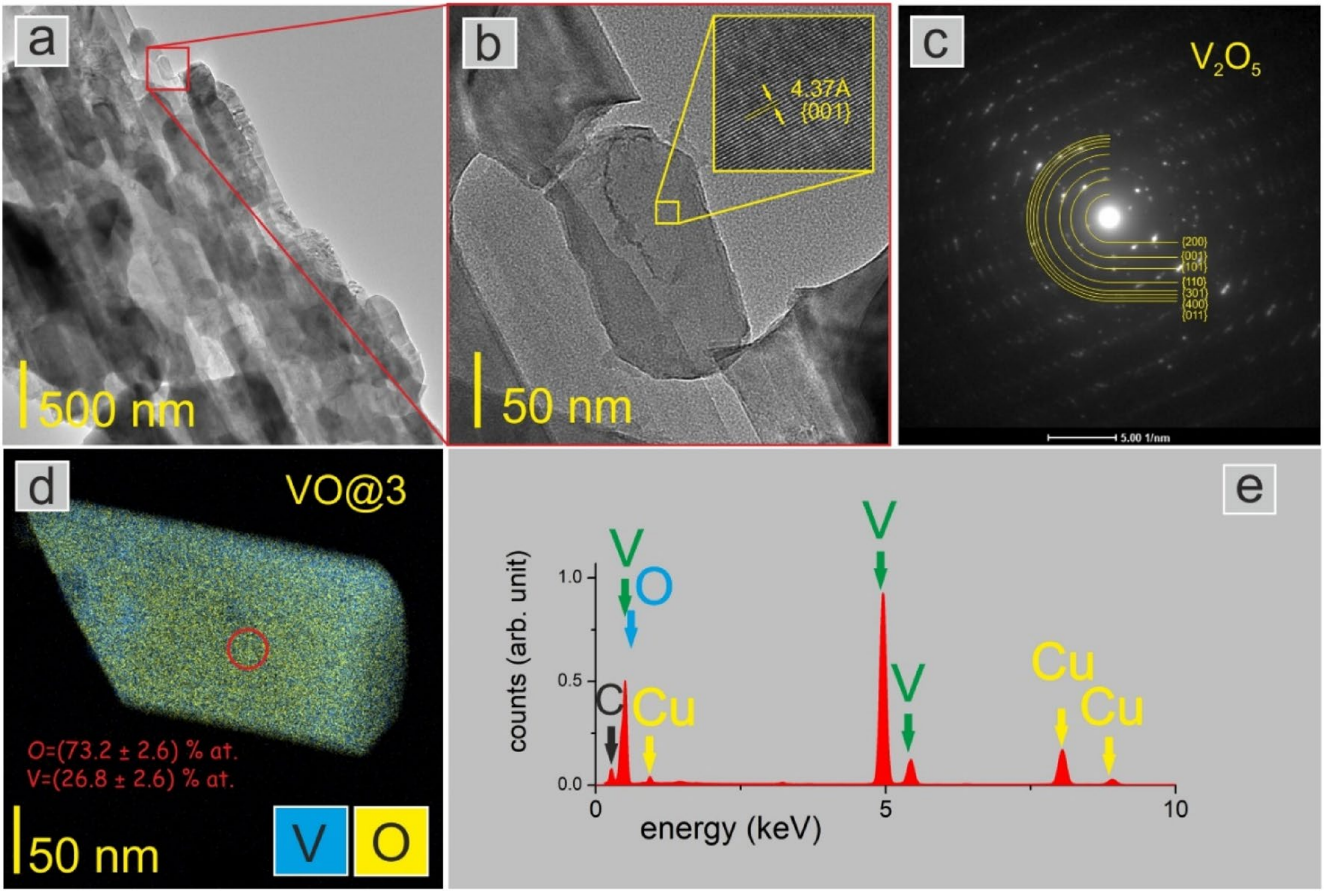
(**a**)TEM image of the polycrystalline nanorod-like structure of VO@3, (**b**) a single nanograin with an inset showing the crystalline structure of the grain, (**c**) an electron diffraction pattern, analysis confirmed the orthorhombic structure for all samples, (**d**) EDX map of a single VO@3 nanograin, chemical analysis confirmed the stoichiometry of the V_2_O_5_ compound, (**e**) EDX spectra collected from the area of the red circle marked on the grain in (**d**). The presence of Cu and C is related to the substrate, a carbon film supported on a copper grid.

Polycrystalline rod-like structures were generally observed in all studied samples. Figure 5a shows an exemplary rod-like structure build of V_2_O_5_ nanograins, in the next Fig. 5b a single nanograin with an inset confirms the crystalline structure.

The crystal structure of all samples was determined and confirmed the results obtained by the XRD method. An example of an electron diffraction pattern with Miller indexes for the orthorhombic crystal structure is presented in Fig. 5c. The created nanorods had a polycrystalline structure that was visible in Dark Field TEM image (see Fig. S1 in Supplementary Information). There are both sphere-like and oval-like particles, like seminanorods. Furthermore, in Fig. S1 the particle size distribution creating such a nanorod is presented for each analyzed sample (VO@1, VO@2, and VO@3). The highest nanorod size (678 nm) was observed for sample VO@3 to which citric acid with ethylene glycol was used. That results are in agreement with XRD studies, where sample VO@3 had the highest average crystallite size of 77 nm. Further examinations will confirm that the best electrochemical performances reveal Cell#3, constructed of sample VO@3.

The chemical analysis of single grains (Fig. 5d) was also performed, and the atomic composition was determined, obtaining the results consistent with the stoichiometry of the V_2_O_5_ compound. It should be added that in individual grains no precipitates from the reagents were observed, and the EDX spectrum collected from the grain—a red circle marked and presented in Fig. 5e showed only the presence of V and O. A surface area of 5.8 m^2^ g^−1^, 5.4 m^2^ g^−1^ and 4.2 m^2^ g^−1^ was observed from the multipoint BET measurement for VO@1, VO@2 and VO@3 respectively.

### Electrochemical studies

Symmetrical configurations of the supercapacitor cells with vanadium dioxide based electrode materials with polymer gel electrolytes are given below:

- Cell#A: VO@1|PVdF-HFP-PC-Mg(ClO_4_)_2_|VO@1
- Cell#B: VO@2|PVdF-HFP-PC-Mg(ClO_4_)_2_|VO@2
- Cell#C: VO@3|PVdF-HFP-PC-Mg(ClO_4_)_2_|VO@3

The performance characteristics of these cells were carried out by using electrochemical impedance spectroscopy, cyclic voltammetry, and the galvanostatic charge–discharge technique, and the results are discussed in the following section.

**Figure 6:**
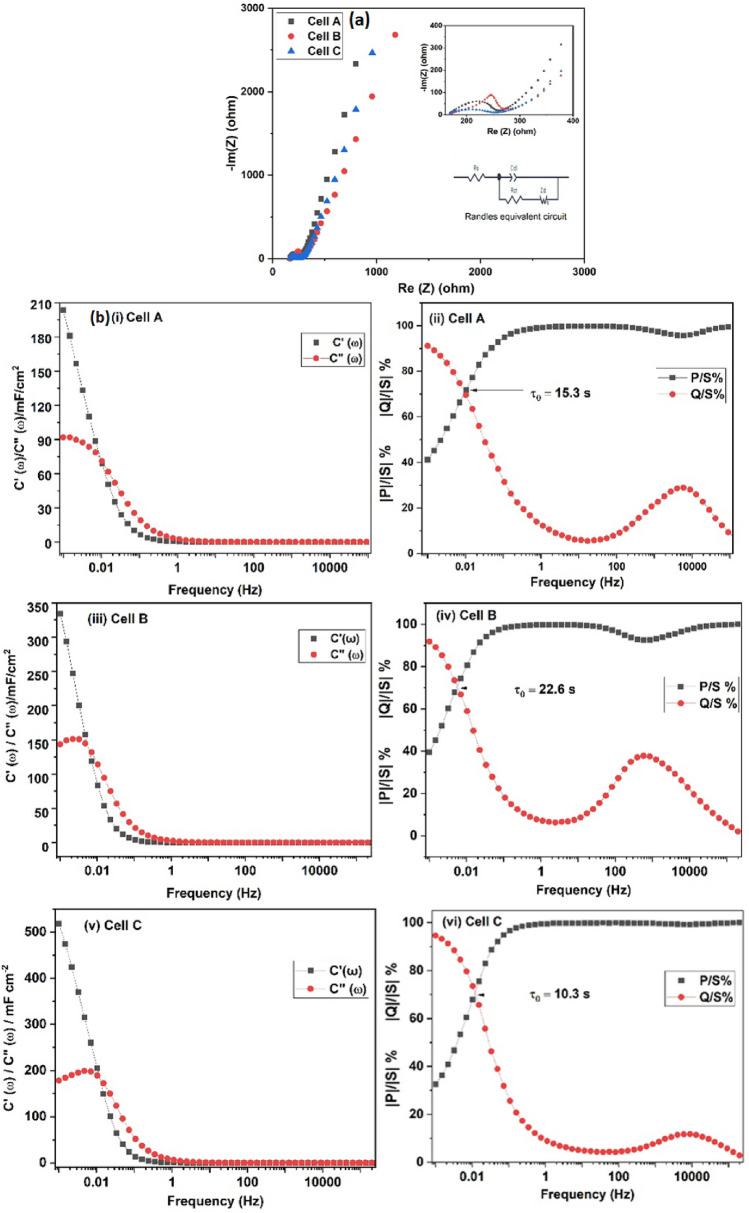
(**a**) EIS plot of Cell #A–#C at 1 mHz recorded at room temperature, (**b**): (**i**): Bode plots for the real and imaginary part of complex capacitance as a function of frequency (in logarithmic scale), (**ii**): normalized active power |P|/|S| and reactive power |Q|/|S| as a function of frequency (in logarithmic scale) for Cell #A, (**iii**): Bode plots for the real and imaginary part of complex capacitance as a function of frequency (in logarithmic scale), (**iv**): normalized active power |P|/|S| and reactive power |Q|/|S| as a function of frequency (in logarithmic scale) for Cell #B, (**v**): Bode plots for the real and imaginary part of complex capacitance as a function of frequency (in logarithmic scale), (**vi**): normalized active power |P|/|S| and reactive power |Q|/|S| as a function of frequency (in logarithmic scale) for Cell #C.

The electrochemical performances of the materials were tested by using polymer gel electrolyte films comprising the composition; PVdF(HFP)-PC-Mg(ClO_4_)_2_. The EIS curves of symmetrical supercapacitor cells (Cell #A, #B and #C) fabricated by using VO@1, VO@2 and VO@3 are shown in Fig. 6a for the frequency range of 10^5^ to 10^–3^ Hz. As can be seen from the figure, the EIS curve for each cell represents a well-defined semi-circular pattern in the high-frequency region followed by steep rising capacitive patterns in the middle to low-frequency regions. The bulk resistance (R_b_) values of cells (R_b_ is mainly due to the combination of electrolyte resistance and intrinsic resistance of active electrode materials^42,43^ and the diameter of the arc refers to charge transfer resistance (R_ct_) that occurred mainly due to charge transfer of the electrode and electrolyte interface are calculated from the semicircle intercept on the real Z axis (expanded view is shown in the inset of Fig. 6a)^44^. The bulk resistance, charge transfer resistance, overall resistance and capacitance values calculated at 1 mHz are compiled in Table 2. The specific capacitance of the capacitor cells was evaluated by using the equation *C*_*s*_ = 2/(2*πf* × *mZ*″), where m is the mass of a single electrode f is the frequency (1 mHz in the present case) and Z″ is the imaginary impedance of the cells at low frequency. It is interesting to note that the bulk resistance values of all of the cells are quite low and almost equal for all of the cells, which confirms the high ionic conductivity of the electrolyte. Furthermore, the R_ct_ value of Cell #C is significantly low, confirming that the charge transfer rate at the electrode–electrolyte interface due to redox activities is as fast as the charge transport in the double-layer capacitance^45^. The equivalent Randle’s circuit is shown in the inset of Fig. 6a, where R_b_ is the bulk resistance of the electrolyte (semicircle in the high-frequency region), furthermore the parallel combination of R_ct_ and pseudocapacitance exists in the mid-frequency region^46,47^ and a linear region inclined at an angle of ~ 45° is attributed to the Warburg impedance element (W)^48–50^. In the circuit, C_dl_ is the capacitance observed at the electrode–electrolyte interface as a straight line in the low-frequency region^51,52^.

**Table 2:** Electrical parameters of supercapacitor cells from EIS analysis.

Cells	R_ct_ (Ω cm^2^)	R_b_ (Ω cm^2^)	1 mHz		
			R (Ω cm^2^)	C	
				(mF cm^−2^)^a^	(F g^−1^)^b^
#A	30.3	18.7	293	36.9	163.9
#B	44.1	13.4	172	46.5	206.7
#C	6.2	13.5	94.3	59.5	264.5

The values of real (C′) and imaginary (C″) capacitance have also been calculated from the frequency-dependent real (Z′) and imaginary (Z″) impedances by using the following equations^43^:

1 $${C}^{{\prime}}\, (\omega )= \frac{-Z"(\omega )}{\omega Z{(\omega )}^{2}}$$

2 $${C}^{"}(\omega )= \frac{Z{^{\prime}}(\omega )}{\omega Z{(\omega )}^{2}}$$

In addition, the rate performance, pulse power, and knee frequency of the cell were evaluated by Bode plots and are shown in Fig. 6b. The point at which the Z′ and Z″ intersect indicates the resonant frequency f_0_ (0.01 Hz, 0.04 Hz and 0.02 Hz respectively for Cell #A–#C). The response time that corresponds to this frequency is ~ 15.3 s, 22.6 s and 10.3 s which is accepted and relatively smaller compared to commercially available supercapacitors^53–55^. At low frequencies, the behaviour of Z′ highly depends on the electrode/electrolyte interface^56^. In the present study, the steep rise at low frequencies clearly confirms the good electrode/electrolyte interface contact resulting in improved access of the electrode material to the electrolyte ions.

**Figure 7:**
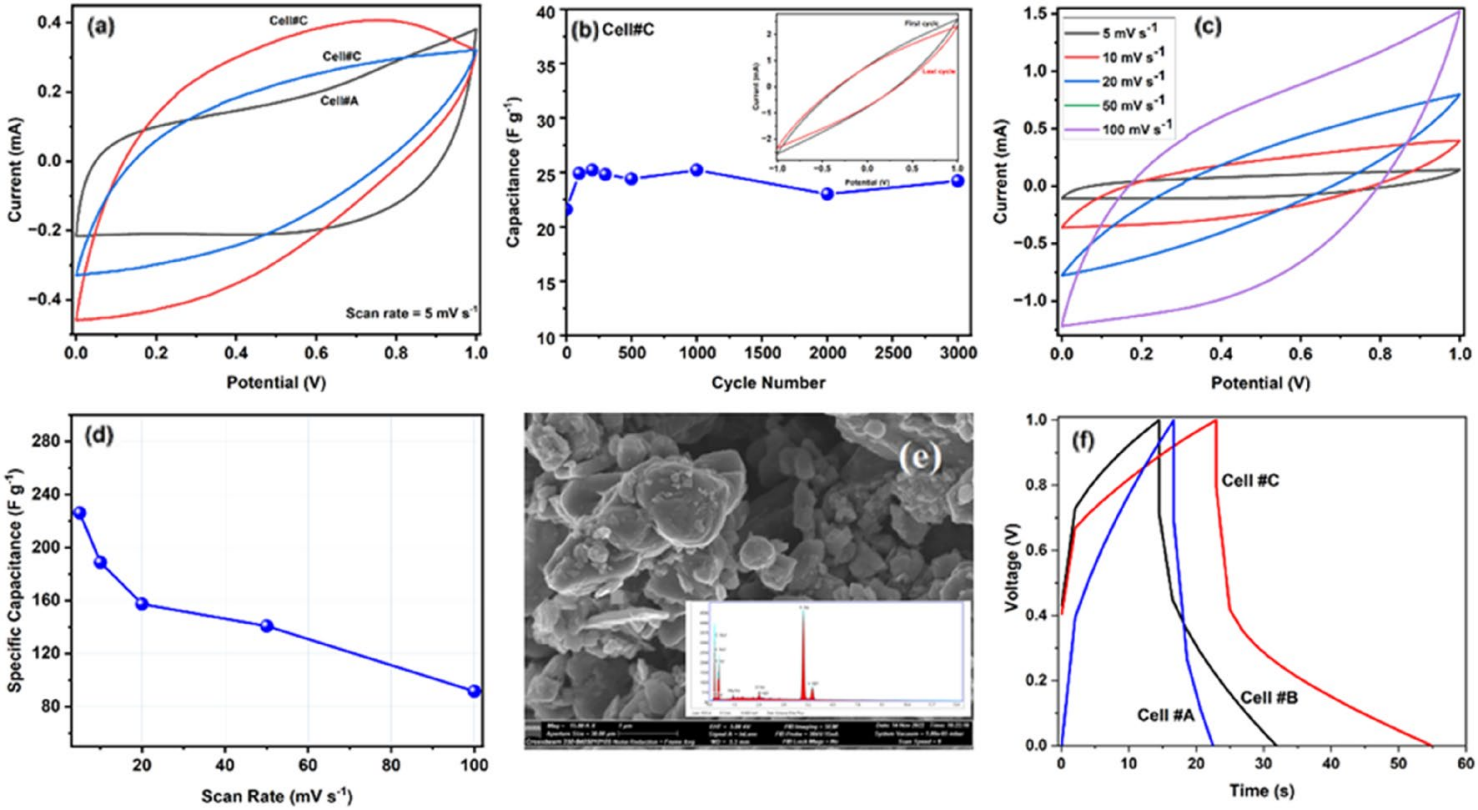
(**a**) Comparative CV curves of the capacitor cells (Cell#A–Cell#C) at a scan rate of 5 mV s^−1^, (**b**) Variation of specific capacitance of Cell#C as a function of cyclic voltammetric cycles at a scan rate of 150 mV s^−1^. The inset shows the first and last CV curves of Cell#C, (**c**) CV curves of Cell #C at different scan rates, (**d**) Variation of specific capacitance of Cell#C with respect to scan rate, (**e**) SEM–EDX spectra of VO@3 electrode after cycling (**f**) GCD curves of Cell#A-Cell #C recorded at a density of 0.56 A g^−1^ at room temperature.

To investigate further the electrochemical properties of the cells, CV tests were also recorded for all cells. As can be seen in Fig. 7a, the CV curves of Cell #A–#C at the scan rate of 5 mV s^−1^ and working potential window of 0–1.0 V shows high capacity. The CV curve shows the mixture of pseudocapacitor behaviour and double layer capacitance. Furthermore, a broad redox peak can also be observed in the CV curves, which corresponds to the electrochemical redox reaction of V^5+^ to V^4+^ ions. The capacitance values from CV studies was calculated by using the formula *C*sp = 2[*ms*(*V*_a_ − *V*_b_)]^−1^ ∫ *I*(*V*) *dV*, where m is the mass of the single electrode, s is the scan rate, (*V*_a_ − *V*_b_) refers to the working potential range^57,58^. The capacitance value of the CV studies were observed to be on the order of 149.2, 137.5, 213.8 F g^−1^ for cell #A to #C respectively at a scan rate of 5 mV s^−1^.

Cycling stability tests are another parameter to evaluate any material for commercial use. The cyclic stability of Cell #C was also tested by evaluating the values of specific capacitance. Cyclic testing was carried out by repeating the cyclic voltammograms for several hundreds of cycles. Figure 7b shows the cyclic testing curve of Cell #C and the inset shows the first and last cyclic voltammogram curves. According to previous studies reported, the capacitance retention of vanadium oxide is not that good in capacitance retention, which may be due to the structural disturbance in the V_2_O_5_ electrode due to the insertion and desertion of electrolyte ions. In the present work ~ 90% of the initial capacitance was retained after ~ 3000 cycles. Upon the cycling, the capacitance fading is mainly due to the mechanical disruption of the electrodes that occurs as a result of repeated swelling or shirking of active materials during the cyclic voltammogram testing process. Rate performance of Cell#C has been investigated by recording the CV curves at different scan rates and is shown in Fig. 7c. As can be seen from the figure, the cell maintains its shapes even at higher scan rates, which confirms the good rate performance of the fabricated cell. The specific capacitance values were also calculated for the cell at different scan rates and are plotted against scan rates and shown in Fig. 7d. As can be seen from the graph, with increasing scan rate, the specific capacitance values decrease, which may be probably due to the insufficient time available for the redox reaction (in and out) reaction inside the bulk part of the electrode material^54^.

To obtain more information, SEM images with EDX pattern of cycled Cell#C electrodes was carried out and is shown in Fig. 7e, it can be clearly seen that after 3000 cycles also, the structure of the electrodes are not changed which confirms the stability of the electrode material, however we can clearly observe some spheres which may be are from electrolyte materials and from EDX studies, we confirmed the presence of Mg^2+^ ions, F^−^ ions, and also ions of oxygen which can be because of the interaction between electrode and electrolyte ions and also these after cycling these big ions block the pathway thereby deteriorating the performance of the device. To further evaluate the electrochemical performance of the vanadium oxide nanorod electrodes, galvanostatic charge–discharge (GCD) measurements were carried out at the current density of 0.56 A g^−1^. The GCD curves of the two-electrode set-up are shown in Fig. 7f, and from the curve it can be seen that it is non-linear in shape, which may be because of the pseudocapacitive behaviour of the electrode material, which was also confirmed by CV studies. The discharge capacitance (C_d_) has been calculated from the linear part of the discharge characteristics using the following equation: , ${C}_{d}= \frac{i x \int Vdt}{2m}$ where i is the constant current density for charge–discharge, $\int Vdt$ is the area of the discharge curve corresponding to the voltage range of ΔV and m is the mass of the active material in a single electrode. Typical values of specific capacitance from GCD studies for cell #A–#C were recorded as ~ 21.3, 67.6 and 141.8 F g^−1^, the cells are evaluated at the current density of 0.56 A g^−1^. From GCD studies, two more important parameters, energy density (E = 1/2* CV*^2^ ) where E (Wh/kg) represents the specific energy, C is the specific capacitance, V is the potential window and powder density (P = *E*/Δ*t*), where P (kW/kg) is the specific power^59,60^. The estimated energy densities are found to be on the order of ~ 2.8, 9.1 and 19.1 Wh kg^−1^, power densities of ~ 1.7, 1.9 and 2.3 kW kg^−1^, respectively. Furthermore, the comparison of electrochemical performance of V_2_O_5_ electrodes with recently reported supercapacitor devices are listed in Table 3. It should also be noted here that the electrochemical properties were strongly dependent on the structure and morphology of the created V_2_O_5_ nanorods, which in turn were influenced by the conditions of the proposed modified sol–gel synthesis, in particular the complexing agents that were used. Specifically, taking into account the characteristic parameters, like the average crystallite size (XRD) and the particle size distribution that created the nanorods (TEM), one can observe that with increasing both analyzed parameters, the best electrochemical performances were achieved. Taking into account, as mentioned previously, the appropriate selection of complexing agents used to the proposed modified sol–gel method of V_2_O_5_ material to form it in the nanorods shape had a huge influence. Our studies revealed that the best electrochemical performances were obtained with Cell#3, constructed of VO@3 sample (synthesized using citric acid and ethylene glycol as a chelating agents). Meanwhile, the VO@3 sample had the lowest specific surface area. Similar behavior between the specific surface area and electrochemical performances we reported in our previous work on the utilization of Co_3_O_4_ material as anode material in lithium-ion batteries^61^. This work and our previous one^61^ confirmed this relationship that the synthesis conditions of the material have a high impact on electrochemical performances.

**Table 3:** Electrochemical comparison of present work with recent supercapacitor devices.

Material	Electrolyte	C_sp_ (F/g) Two electrode system	Energy density (Wh kg^−1^)	References
V_2_O_5_/PANI/Graphene	1 M Na_2_SO_4_	127	57	^62^
V_2_O_5_@N-MWCNT/CMC	1 M H_2_SO_4_	138	38.8	^63^
V_2_O_5_/VACNTs	1 M Na_2_SO_4_	284	32.3	^64^
V_2_O_5_/AC	PVA-LiClO_4_	137	27.4	^65^
Mesoporous carbon microspheres/V_2_O_5_	1 M Al_2_(SO_4_)_3_	290	18.0	^25^
V_2_O_5_-PANI/NiMn_2_O_4_	1 M H_2_SO_4_	71.2	25.3	^66^
V_2_O_5_ nanorods	PVDF-HFP-PC-Mg(ClO_4_)_2_	141.8	19.1	Present work

The coulombic efficiency, which is the ratio of charging time to the discharging time is also one of the important parameter related with the charge–discharge behavior of supercapacitors. The coulombic efficiency calculated by using the equation η = *t*_*D*_/*t*_*C*_ × 100%, where t_D_ and t_C_ are the discharging and charging time of the supercapacitor cell. The coulombic efficiency calculated by using the above formula is found to be of the order of ~ 80% which confirms the liquid like nature of polymer electrolyte used in the present studies.

## Conclusions

Vanadium oxide nanorods were synthesized by using a modified sol–gel technique with different chelating agents. Vanadium oxide prepared using ethylene glycol as a chelating agent achieved the best results and the electrodes exhibit a specific capacitance of ~ 141.8 F g^−1^ with a power density of ~ 2.3 kW kg^−1^ and an energy density of ~ 19.1 Wh kg^−1^. The cells showed excellent rate capability and good cycling stability up to ~ 3000 voltametric cycles. The superior capacitive performance of the electrode was mainly due to the nanorod structure, which was confirmed from TEM studies. According to the presented studies, the synthesized V_2_O_5_ material can be considered as a potential candidate for energy storage devices.

## Supplementary Information

Supplementary Figure S1.

## Data Availability

The data are available on the link: 10.6084/m9.figshare.21155236.
