# Supplementary material for: Vanadium oxide nanorods as an electrode material for solid state supercapacitor
Source: Sci Rep. 2022 Dec 5;12:21024. doi: 10.1038/s41598-022-25707-z (PMC9723181; doi:10.1038/s41598-022-25707-z)
Supplement: Supplementary file 1 — Supplementary Figure S1. [file 41598_2022_25707_MOESM1_ESM.pdf]

Supplementary Information

Vanadium oxide nanorods as an electrode material for solid state supercapacitor

Amrita Jain[1], Sai Rashmi Manippady[1], Rui Tang[2], Hirotomo Nishihara[2,3], Kamil Sobczak[4], Vlastimil Matejka[5], Monika Michalska[5*]

[1]Institute of Fundamental Technological Research, Polish Academy of Sciences, Pawińskiego 5B, 02-106 Warsaw, Poland

[2]Advanced Institute for Materials Research (AIMR-WPI), Tohoku University, 2-1-1 Katahira, Aoba-ku, Sendai 980-8577, Japan

[3]Institute of Multidisciplinary Research for Advanced Materials, Tohoku University, 2-1-1 Katahira, Aoba-ku, Sendai 980-8577, Japan

[4]Faculty of Chemistry, Biological and Chemical Research Centre, University of Warsaw, Zwirki i Wigury 101, 02-089 Warsaw, Poland

[5]Department of Chemistry and Physico-Chemical Processes, Faculty of Materials Science and Technology, VŠB-Technical University of Ostrava, 17. listopadu 2172/15, 708 00 Ostrava-Poruba, Czech Republic

*Corresponding author: monika.kinga.michalska@gmail.com

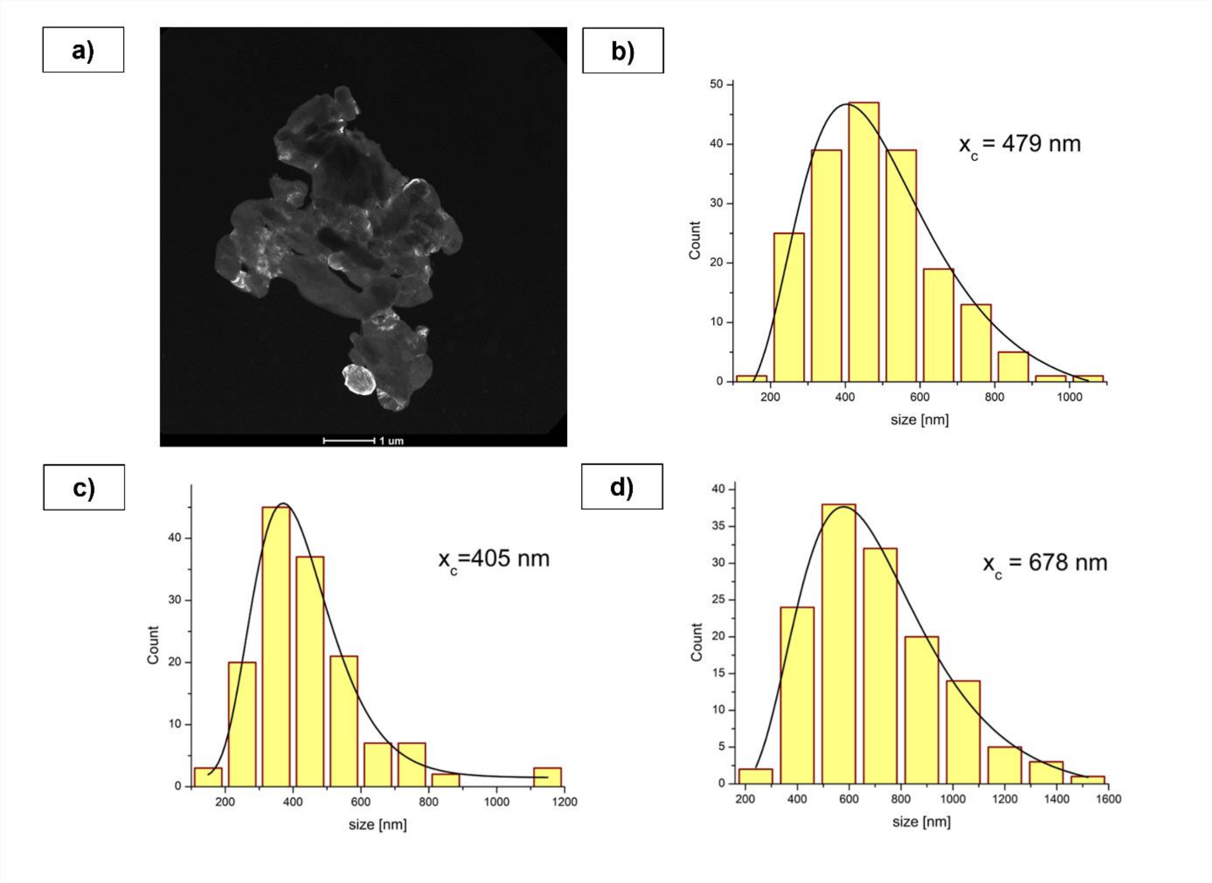

Figure S1. Dark Field TEM image of V_2O_5 material (sample @VO3) (**a**), and size distribution of NPS that creates nanorods for samples: VO@1 (**b**), VO@2 (**c**), and VO@3 (**d**).
